# Probiotic potential of *Lactobacillus plantarum* DMR14 for preserving and extending shelf life of fruits and fruit juice

**DOI:** 10.1016/j.heliyon.2023.e17382

**Published:** 2023-06-19

**Authors:** Shirmin Islam, Suvro Biswas, Tabassum Jabin, Md. Moniruzzaman, Jui Biswas, Md. Salah Uddin, Md. Akhtar-E-Ekram, Abdallah M. Elgorban, Gajanan Ghodake, Asad Syed, Md. Abu Saleh, Shahriar Zaman

**Affiliations:** a Microbiology Laboratory, Department of Genetic Engineering and Biotechnology, University of Rajshahi, Rajshahi, 6205, Bangladesh; b Department of Botany and Microbiology, College of Science, King Saud University, P.O. Box 2455, Riyadh, 11451, Saudi Arabia; c Department of Biological and Environmental Science, Dongguk University-Seoul, Ilsandong-gu, Goyang-si, 10326, Gyeonggi-do, South Korea

**Keywords:** Lactic acid bacteria, Antimicrobial activity, Probiotic potential, Preservation, Shelf life

## Abstract

The harmful effects of chemical preservatives are driving the need for natural ones. To meet this demand, probiotic lactic acid bacteria (LAB) were isolated from fermented oats in this study. The goals of this study were to separate and identify probiotic LAB from fermented oats, to determine how effective these LAB are at combating pathogenic microorganisms in vitro, and to investigate their preservative capacity by applying the bacterium's cell-free supernatant (CFS) to specific fruits and fruit juice. The isolated strain was identified as *Lactobacillus plantarum* DMR14 using morphological, biochemical, and molecular investigation. Antimicrobial, antibiofilm, anti-oxidant, pH tolerance, and antibiotic resistance assays were used to evaluate the strain's probiotic potential, showing that *Lactobacillus plantarum* DMR14 had the strongest antagonistic and *anti*-biofilm capacity against *Shigella boydii*. Furthermore, the bacteriocin-containing compounds, cell-free supernatant (CFS) of the LAB, were tested against three fruits and one fruit juice, with the cell-free supernatant (CFS) of the bacterium lengthening the shelf life of the fruits compared to the untreated ones. Furthermore, while the concentration of coliform bacteria decreased in the treated sugarcane juice, an increase in the concentration of lactic acid bacteria suggested that the strain may be used as a fruit preservative in food industries.

## 1 Introduction

At present, a wide range of physical techniques and chemical reagents are employed for fruit and fruit juice preservation processes. These preservation methods aim to prevent or delay the spoilage of fruits and fruit juices caused by microbial activity, oxidation, or enzymatic degradation. Physical techniques, such as refrigeration, freezing, and dehydration, are frequently used to prolong the shelf life of fruits and juices. Chemical reagents, such as antioxidants, preservatives, and antimicrobial agents, are also commonly used to preserve fruits and juices. However, the use of these methods may have drawbacks, such as changes in the nutritional and sensory qualities of the preserved products [1,2]. Apart from their ability to regulate unwanted microorganisms, traditional techniques such as filtration or thermal processing may also negatively impact the natural components of biological products, such as aromatic compounds, flavoring ingredients, and other compounds. This means that while these methods are effective in preserving food, they may alter its overall quality and flavor [3]. There are several chemical substitutes that can be used as food preservatives, and among them, the most commonly used ones are chlorine-based compounds [4]. However, alternative methods have also been developed to achieve similar results. For instance, organic acids, ozone, peroxyacetic acid, electrolyzed water, hydrogen peroxide, and calcium-based solutions [5] have been found to be effective in preserving food and preventing spoilage. These substitutes offer diverse options for food manufacturers to choose from, depending on the specific needs and requirements of their products. Consumer concern over food safety has generally increased due to the widespread misuse of chemical synthetic food preservatives, which has prompted a search for natural and safe food preservatives to replace the chemical synthetic ones [6]. Natural antibacterial agents may be used effectively to preserve food and maintain its quality and safety. Consumers are progressively becoming more interested [7] in the innovative and alternative techniques of food bio preservation [8]. Fresh-cut fruits and vegetables have a very short shelf life and experience severe physiological deterioration, and biochemical changes and microbiological degradation may occur while selling. All of these changes can cause considerable loss of sensory attributes, such as color, smell, and flavor, as well as the development of harmful and dangerous organisms, resulting in a decrease in shelf life [7,9]. Biopreservation is a natural and regulated process that can help ensure the safety of fresh fruits and vegetables, making them a healthier and more sustainable option for consumers. Fresh fruits and vegetables, including those that have been freshly cut, can be preserved using biopreservation methods, which extend their shelf life by utilizing natural and safe microflora without any harmful biologically active substances. This technique involves introducing beneficial bacteria, such as lactic acid bacteria or other probiotics that can prevent the growth of harmful microorganisms while also maintaining the quality and freshness of the produce [10,11]. Probiotics, which are living bacterial species that are beneficial to human health, are being utilized in the food industry as preservatives to prevent the growth of harmful microorganisms and as potential substitutes for antibiotics to prevent or treat various diseases [12]. The majority of probiotics used in food products belong to the group of lactic acid bacteria (LAB), which includes *Bifidobacterium* and *Lactobacillus.* These bacteria are capable of producing several active metabolites, such as bacteriocins, reuterin, and hydrogen peroxide, which are low molecular mass compounds that can effectively combat harmful microbes. Moreover, these active compounds produced by probiotics are safe, natural, and have no adverse effects on human health, making them a healthier and more sustainable alternative to traditional preservatives and antibiotics [13,14]. In addition to their ability to produce active metabolites that combat harmful microorganisms, LAB is also classified as “Generally Recognized As Safe” (GRAS) by the US Food and Drug Administration (FDA) due to their immunomodulatory, antibacterial, *anti*-biofilm, and antioxidant properties. These beneficial properties of LAB make them an attractive alternative to synthetic food preservatives and antibiotics, as they offer a natural and safe solution for food preservation and promoting human health. Furthermore, the GRAS status of LAB highlights their widespread acceptance and use in the food industry, indicating their potential for future development and applications in food technology [15].

Lactic acid bacteria can be obtained from various sources, but dairy products are considered the most viable and suitable source for probiotics that offer potential health benefits to consumers [16,17]. Despite this, the high sugar content, cholesterol levels, and lactose intolerance issues associated with dairy products have hindered the development of dairy-based probiotic products. Therefore, alternative sources of lactic acid bacteria are being explored, such as plant-based sources like fermented vegetables, fruits, and grains. These non-dairy sources offer a more sustainable and healthier option for probiotic food products, which can cater to a wider range of consumers with different dietary restrictions or preferences [18,19]. Table 1 depicts several cereal-based sources of lactic acid bacteria from previous studies, indicating that cereal-based foods may be an enriched source of probiotic lactic acid bacteria.

**Table 1:** Cereal-based foods sources of lactic acid bacteria.

Cereal based foods	Name of the lactic acid bacteria	References
Rice	*Lactobacillus brevis, Lactobacillu curvatus, Lactobacillus plantarum* DMR14*, Lactobacillus farciminis* D323	[[20], [21], [22]]
Maize	*Lactobacillu fermentum*, *Lactobacillu rhamnosus*, *Lactococcus*, *Pediococcus*	[[23], [24], [25]]
Sorghum	*Lactobacillus. delbrueckii, Lactobacillu, fermentum*	[26]
Wheat	Lactobacillus coryniformis, *Leuconostoc mesenteroides, Leuconostoc mesenteroides, Lactobacillus brevis, Lactobacillus. Acidophilus*	[[27], [28], [29]]
Rye	*Weissella paramesenteroides, Lactobacillus, Lactobacillus. Kimchi, Lactobacillus plantarum* DMR14*, Lactobacillus brevis*	[30,31]
Barley	*Lactobacillus* spp. *Pediococcus acidilactici, Enterococcus faecalis, Lactobacillus plantarum* DMR14	[32]
Oat	*Lactobacillus plantarum* DMR14*, Streptococcus thermophilus DSM 20259, Lactobacillus delbrueckii* subsp. *bulgaricus NCFB* 2772	[33,34]
Chia	*Lc. Lactis, Lactobacillus plantarum* DMR14	[35]
Millet	*Lactobacillu. fermentum, , Lactobacillus* spp.	[36,37]

To explore alternative sources of lactic acid bacteria for use as probiotics and natural preservatives, the study aimed to isolate new probiotics from one of the most commonly consumed cereal-based foods. In addition to identifying a new source of lactic acid bacteria, the study aimed to assess the probiotic potential of the isolated strain and its ability to naturally preserve fruits and fruit juices, offering a more sustainable and eco-friendly preservation technique. The study's findings suggest that the isolated strain has the potential to be used as a probiotic and natural preservative, providing a promising alternative to conventional preservatives that are associated with adverse health and environmental effects. By utilizing natural and safe microflora, this approach could promote healthier and more sustainable food preservation practices.

## 2 Materials and method

### 2.1 Sample collection and isolation of the bacteria

Quaker oats samples were collected from a super shop of Shaheb-bazar Rajshahi, Bangladesh, and brought to the microbiology laboratory, department of Genetic Engineering and Biotechnology, University of Rajshahi, where 5 g of oats sample was fermented overnight.

LAB were isolated on MRS agar after incubation with MRS broth for 12 h at 37 °C. Plates were then incubated at 37 °C for 24 h aerobically. An isolated strain was sub-cultured in MRS broth. Glycerol stocks (50% v/v) were prepared for each colony and stored at −80 °C.

### 2.2 Molecular analysis

According to the procedure described by Naeem et al. [38], the genomic DNA of the bacteria in its pure form was isolated. To achieve this, universal forward and reverse primers, specifically 1492R (5′- GGTTACCTTGTTACGACTT-3′) and 27F (5′-AGAGTTTGATCCTGGCTCAG-3′), were employed. The amplified products from the PCR process were then submitted for 16S rRNA-sequencing to a commercial sequencing service (Invent Technologies Ltd. Dhaka, Bangladesh).

### 2.3 Phylogenetic analysis

Using BLAST and the Gene Bank Internet service, it was possible to identify the bacterial strains down to the species level. The 16S rRNA sequence data was then uploaded to the GenBank database (https://submit.ncbi.nlm.nih.gov) and MEGA-X software was used to carry out the phylogenetic and molecular evolutionary study.

### 2.4 Probiotic properties

The chosen bacterial strain underwent additional screening to determine its probiotic qualities. The LAB species was employed, and the probiotic qualities of the species were carefully examined. The bacterial strain needs to successfully complete each of the tests required at this stage, in accordance with FAO/WHO recommendations, in order to establish its viability as a possible probiotic candidate (https://www.fao.org/3/a0512e/a0512e.pdf). This screening employs a number of in vitro tests, which are described in more details in the following sections.

#### 2.4.1 Acid tolerance

Acid tolerance of the chosen bacterial strain was investigated. The medium used was MRS broth containing 3 mg/mL of pepsin enzyme. 1.0 N HCl was used to adjust the pH of broth at various pH values (2.0, 3.0, 4.0, 5.0, 6.0, 7.0, and 8.0), and the same solution was used as a control set (pH 7.0) [39]. The broth was infused with overnight-grown cultures of potential strains and was then incubated for 24 h at 37 °C. The sampling process was performed every 12 h. At 620 nm, the optical density was determined, and viable counts were also taken into account.

#### 2.4.2 Bile salt tolerance

This test was performed using oxgall (HiMedia) at a concentration of 0.3% (w/v)). The isolated overnight cultured strain was then spread on it and the presence and absence of the bacteria was observed.

#### 2.4.3 Antibiotic susceptibility (Kirby-Bauer) test

The technique employed to obtain information on the resistance of bacterial isolates to antibiotics was the Kirby-Bauer disc diffusion method with some modifications [40]. This technique categorized the obtained results as either susceptible, intermediate resistance or resistant. The interpretation of the zone diameters was done using the performance standards for antimicrobial disk susceptibility tests.

#### 2.4.4 Antioxidant capacity of intact cells

##### 2.4.4.1 Preparation of intact cells

To obtain intact cells, a 24-h broth culture was prepared and then centrifuged at 10,000 rpm for 10 min. The resulting cell density was adjusted to 10^8^ CFU/mL using MacFarland 0.5. This adjustment was made using 0.85% saline.

##### 2.4.4.2 DPPH test

The DPPH free radical method was used to observe the antioxidant activity of the chosen strain. This method was modified according to the description provided by Yang et al. [41. To summarize, the experiment involved mixing 2 mL of whole cells with 1 mL of methanolic DPPH solution and incubating them for 30 min in the absence of light. After this, the solutions were subjected to centrifugation at 6000 rpm for 10 min. Anhydrous methanol was used as a blank, while a mixture of 2 mL of methanol and 1 mL of DPPH was employed as a control. Optical density measurements were taken at 517 nm, and the scavenging activity was calculated using a specific formula. The GENSYS 10S UV-VIS Thermo Scientific Spectrophotometer was utilized to determine the OD value. The following equation was used to calculate antioxidant percentages:

Scavengingrate(%)=1−(sample−blank)control×100%

### 2.5 Screening of lipolytic activity

Tween 80 media (Oxoid Ltd., Basingstoke Hampshire, UK) was used for the evaluation of lipase activity according to established protocol [41]. Briefly, tween 80 was added to TSA at a ratio of 1:100. Then, phenol red (Sigma-Aldrich, St. Louis, MO, USA) was added as an indicator. The LAB was then streaked on the plate and incubated for 24–48 h at 37 °C. Color change from red to yellow–orange indicated positive results.

### 2.6 Screening of amylolytic activity

2.5 g of starch agar (Himedia, India) were added to 100 mL distilled water and autoclaved at 121 °C for 15 min. The probiotic strain was then streaked on the media and incubated for 24 h at 37 °C. Then, the media was flooded with 1% iodine solution (Himedia, India) [41].

### 2.7 Screening of hemolytic activity

Overnight cultured LAB was grown and streaked on a blood agar plate (4% sheep) (HiMedia Laboratories, Mumbai, India) and incubated for 48 h at 37 °C. The presence or absence of zones of hydrolysis around the colonies was examined [42].

### 2.8 Antimicrobial tests

The antibacterial activity of the strain of lactic acid bacteria was evaluated through the agar-well diffusion method, which was carried out based on prior literature, with certain adjustments [43]. The strain was examined for its ability to generate antimicrobial substances against the bacteria that were tested (Table S2). Mueller Hinton agar plates were produced and the indicator bacteria were added. Wells with a diameter of 5 mm were created in the agar plates and loaded with 100 μl of the tested strain's culture. The plates were then incubated at 37 °C for 24–48 h. The diameter of the clear zone of inhibition was measured in millimeters.

### 2.9 Antibiofilm tests

#### 2.9.1 Biofilm formation assay

Biofilm formation test was performed following the protocol of**microtiter** plate (MtP) **assay** [44]. Here, the selected bacterial strains (100 μl of each strain) (Table S2) were allowed to grow in wells of a 96-well microtiter plate (Tarsons, India) containing 100 μl of luria bertani (LB) liquid medium and then incubated on an incubator at 37 °C for 24 h without shaking to allow the formation of biofilm at the bottom of the wells. After the incubation period, the plate was rinsed twice with double distilled water, air-dried, and then oven-dried at 37 °C for 1 h (60 min). The biofilm was fixed with 150 μl of absolute methanol for 5 min. After washing, the biofilm was stained with crystal violet (0.1%) for 15 min. Finally, 100 μl of dilution was quantified by measuring at 595 nm.

#### 2.9.2 Inhibition of biofilm formation by cell free supernatants (CFSs)

The previously described protocol (2.9.1) was used to assay the biofilm inhibition ability of the cell free supernatants with some modifications. Here the effect of cell free supernatants (CFSs) of the bacteria on biofilm formation was measured using the co-incubation method. Here, 100 μl CFSs (centrifuged at 12000 rpm for 20 min at 4 °C to obtain cell free supernatants) was added to the bacterial inoculum. Disruption percentage was calculated using the following equation:

DisruptionPercentage=(ODcontrol–ODsample)×100/control

### 2.10 Extending and preserving shelf life of fruit and fruit juice with LAB

#### 2.10.1 Preservation of fruits

The approach proposed by Dhundale et al. was used to investigate the use of LAB in prolonging and maintaining the freshness of fruits [45] with some modifications. The culture was added to 100 mL of MRS broth, which was then incubated for 48 h at 37 °C. After incubation, the broth was centrifuged at 10,000 rpm for 20 min and the resulting supernatant was used as a biofilm coating agent. The fruits in the experimental group were coated with the supernatant after being dipped in it, while the control group consisted of uncoated fruits dipped in sterile distilled water. The coated fruits were dried with air for an hour to eliminate any surface moisture.

#### 2.10.2 Preservation of fruit juice

The locally available sugarcane juice was used to preserve it. The lactic acid bacterium was grown on MRS broth and incubated it at 37 °C for 24 h. After centrifuging at 4000 rpm for 10 min, a 15 mL of CFS was collected. Then, we inoculated 1 mL of CFS into 20 mL of sugarcane juice and incubated it at 37 °C for 48 h. Sampling was performed every 24 h for microbiological and chemical analysis. Viable cells were counted using the standard plate count method with MRS agar and Mac-Conkey agar medium and expressed the results as colony-forming units per milliliter of sample (CFU/mL).

### 2.11 Statistical analysis

Each test was analyzed in duplicate, and the entire experiment was run in triplicate. The outcomes are presented as mean SD (standard deviation). The statistical computer program SPSS 13.0 was used to examine the experimental data (Apache Software Foundation, USA).

## 3 Results

### 3.1 Bacterial isolation

In this study, the LAB was initially cultured on Selective De Man, Rogosa, and Sharpe (MRS) media. The process was maintained in triplicate to ensure consistency. The selection of a pure isolate of the LAB strain was based on observing the colony characteristics of the plate (Fig. S1).

### 3.2 Bacterial identification based upon 16S rRNA sequencing

After the completion of the 16SRrNA gene sequencing, the obtained sequence was compared with the 16S rRNA gene sequences of various organisms present in the NCBI Genebank database. Where, numerous sequences were discovered that exhibited significant similarity to the isolate. Among them, the isolate demonstrated a higher degree of identity (92.24%) to *Lactobacillus plantarum* DMR14.

### 3.3 Phylogenetic position of bacterial isolate

**Fig. 1:**
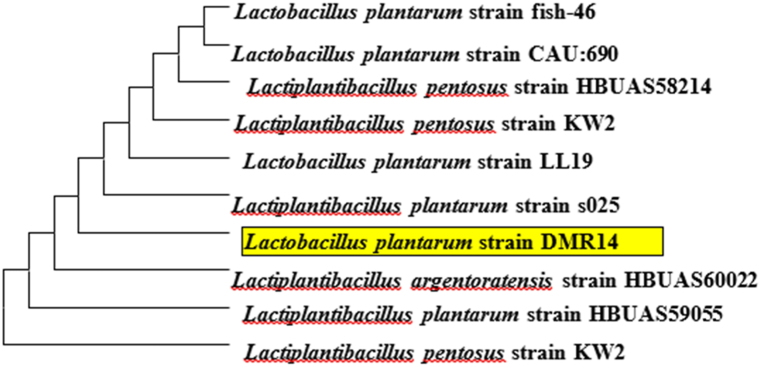
Phylogenetic tree showing the evolutionary relationship of the isolated bacterial strain. A maximum likelihood nearest-neighbor interchange tree was constructed using MEGA 11.

Relationships of the bacterial strain *Lactobacillus plantarum* DMR14 to similar isolates were deciphered using a phylogenetic tree (Fig. 1).

### 3.4 Probiotic properties of the Lactobacillus plantarum DMR14

#### 3.4.1 pH tolerance test

**Fig. 2:**
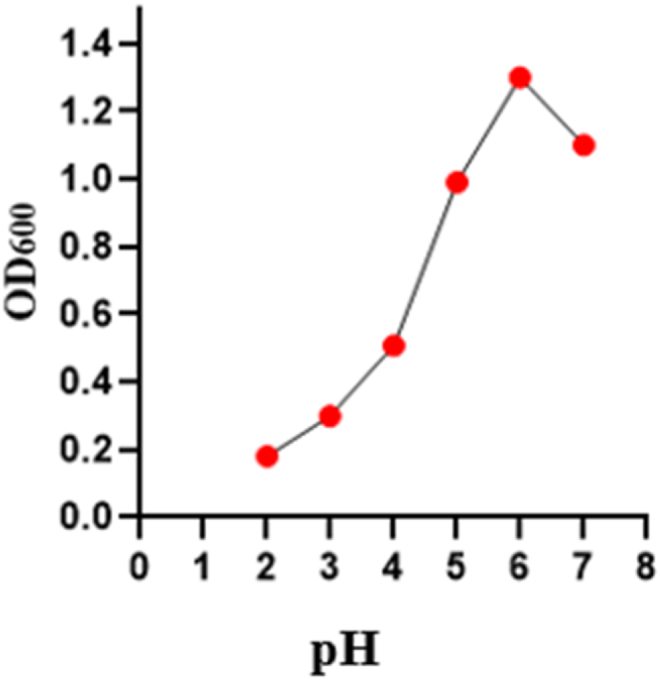
pH tolerance level of the *Lactobacillus plantarum* DMR14. Various pH ranging from 2 to 7 were used. Optical density was measured at 600 nm. Data were recorded after 24 h of incubation.

Acid tolerance of the bacteria was observed at various pH levels ranging from 2 to 7. The optimum pH of the bacteria was 6.0 (Fig. 2).

#### 3.4.2 Bile salt tolerance

In the present study, *Lactobacillus plantarum* DMR14 was able to grow in the presence of bile salts **(**Table 2**)**. Tolerance to bile salts is required for the growth and metabolic activities of bacteria in the intestine of the host.

**Table 2:** Bile salt (0.3%) results of *Lactobacillus plantarum* DMR14.0.3% bile salt was used for this study. Data were recorded 4 different time durations.

Name of the Bacteria	Bile Result (3 h)	Bile Result (6 h)	Bile Result (9 h)	Bile Result (12 h)
***Lactobacillus plantarum* DMR14**	Positive	Positive	Positive	Positive

### 3.5 Antibiotic susceptibility test

An antibiotic sensitivity test was done for the selected strain, with the results shown in Table 3. The strain showed resistance against amoxicillin (AMX) and erythromycin (E), as well as susceptibility to kanamycin (K), gentamycin (G), doxycycline (DO), and chloramphenicol (C).

**Table 3:** Antibiotic sensitivity test result. 9 different antibiotics were used. Here, S indicates sensitive, I indicates intermediate resistant, and R indicates resistant.

Antibiotics Name	Zone of Inhibition (mm)	Status
Kanamycin (K)	20.33 ± 1.52	S
Gentamycin (G)	19.67 ± 2.08	S
Penicillin (P)	11 ± 1	I
Ampicillin (AMP)	10 ± 1	I
Ciprofloxacin (CIP)	28.33 ± 2.88	S
Amoxicillin (AMX)	6	R
Doxycycline (DO)	18 ± 1	S
Chloramphenicol (C)	21 ± 1	S
Erythromycin (E)	6	R

**Note:**Resistant=<10 mm; Intermediate = 10–15 mm; Susceptible=>15 mm.

### 3.6 Antioxidant activity test

An antioxidant test was carried out on the isolate. The result indicated that the strain had 43.6% DPPH scavenging activity (Table 4)**.**

**Table 4:** Antioxidant activity test result of the intact cells of *Lactobacillus plantarum* DMR14.

Bacterial Name	Antioxidant Percentage
*Lactobacillus plantarum DMR14*.	43.6%

### 3.7 Amylolytic, hemolytic, and lipolytic activity tests results

The selected putative probiotic bacterial strain was tested using various in vitro enzymatic potential tests. In this study, *Lactobacillus plantarum* DMR14 showed positive lipolytic, proteolytic, and amylolytic activities (Table 5)**.**

**Table 5:** Lipolytic, amylolytic, and hemolytic activity test results of *Lactobacillus plantarum* DMR14.

Test Name	Result
Amylolytic	Positive
Hemolytic	Gama hemolytic
Lipolytic	Positive

### 3.8 Antagonistic test

*Lactobacillus plantarum* DMR14 displayed varying degrees of antimicrobial activity against six pathogenic bacteria. The bacterium's cell-free supernatant (CFS) effectively suppressed biofilm formation in all bacteria tested. Notably, the highest inhibition rate (67.7%) was observed against *Shigella boydii*. Relatively weaker inhibition activity was observed against *Pseudomonas* sp., *Aeromonas* sp., and *Staphylococcus aureus*, while no significant inhibition was observed against *Escherichia coli* and *Bacillus cereus* (Table 6).

**Table 6:** Antagonistic activity of the *Lactobacillus plantarum* DMR14 against six selected pathogenic bacterial strains.

Bacterial Name	Zone of Inhibition (mm)	Status
*Shigella boydii*	17 ± 1	Moderate/average inhibition (++)
*Pseudomonas* sp*.*	12 ± 1	Weak inhibition (+)
*Escherichia coli*	7.33 ± 0.57	No inhibitory activity (−)
*Bacillus cereus*	6	No inhibitory activity (−)
*Aeromonas* sp.	12.67 ± 1	Weak inhibition (+)
*Staphylococcus aureus*	13 ± 0.57	Weak inhibition (+)

Note: 12–15 mm, weak inhibition (+); 16–19 mm, moderate/average inhibition.

### 3.9 Antibiofilm test

#### 3.9.1 Biofilm formation assay

Biofilm formation is one of the main causes of antibiotic resistance. All bacteria showed strong biofilm formation ability. The biofilm formation abilities of the selected six bacterial strains are shown in Table 7.

**Table 7:** The biofilm formation efficacy of the selected bacterial strains.

Name of the Bacteria	Range	Result
*Shigella boydii*	OD > 4 × ODcutoff	Strong
*Pseudomonas* sp.	OD > 4 × ODcutoff	Strong
*Staphylococcus aureus*	OD > 4 × ODcutoff	Strong
*Escherichia coli*	OD > 4 × ODcutoff	Strong
*Bacillus cereus*	OD > 4 × ODcutoff	Strong
*Aeromonas* sp.	OD > 4 × ODcutoff	Strong

#### 3.9.2 Biofilm inhibition of the LAB CFS

**Fig. 3:**
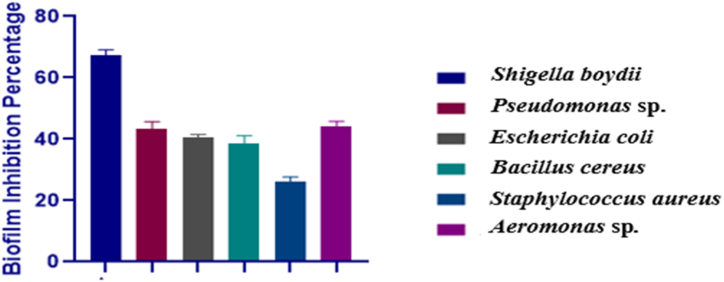
Biofilm inhibition percentage of the cell free supernatants (CFS) of the *Lactobacillus plantarum* DMR14 against the selected bacterial strains. The values were reported in optical density (OD) as mean ± SD of three replicates.

The findings revealed a significant reduction in biofilm formation following treatment with the LAB's cell-free supernatants (CFSs). Among the tested strains, the lowest *anti*-biofilm activity (25.64%) was observed against *Staphylococcus aureus*. Notably, the attachment of *Shigella boydii* to the microtiter plates was markedly inhibited, with a reduction of 67.87%. The biofilm inhibitory potential of the CFSs against bacterial pathogens is visually presented in Fig. 3.

### 3.10 Extending and preserving the shelf life of fruit and fruit juice with Lactobacillus plantarum DMR14

**Fig. 4:**
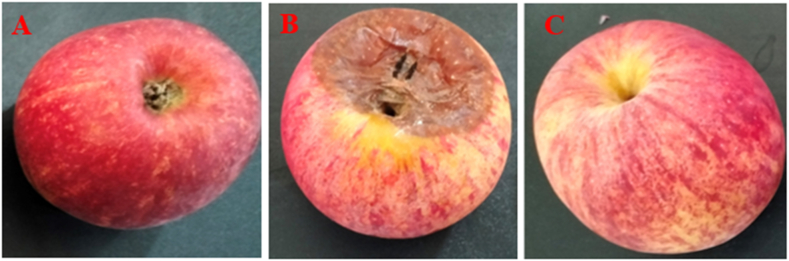
Extending shelf-life of apple with the CFS of *Lactobacillus plantarum* DMR14 (A) Fresh apple at 0 h, (B) after 7 days without treatment; (C) after 7 days with treatment.

**Fig. 5:**
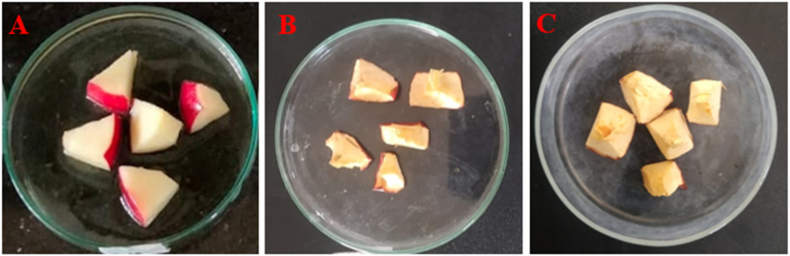
Extending shelf-life of chopped apples with the cell free supernatants (CFS) from *Lactobacillus plantarum* DMR14. (A) Fresh chopped apple at 0 h, (B) and (C) after 3 days without treatment and with treatment, respectively.

**Fig. 6:**
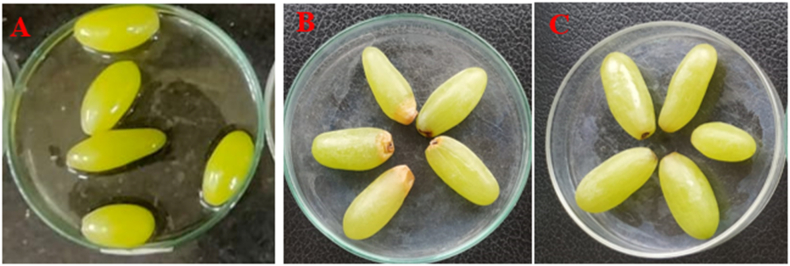
Extending shelf-life of grapes with the cell free supernatants (CFS) of *Lactobacillus plantarum* DMR14. (A) Fresh grapes at 0 h, (B), and (C) after 5 days without treatment and with treatment, respectively.

**Fig. 7:**
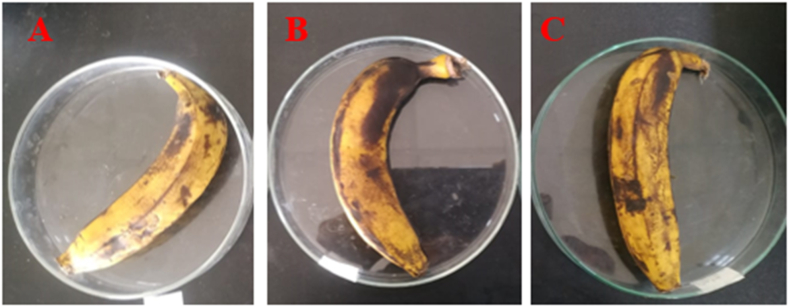
Extending shelf-life of the bananas with the cell free supernatants (CFS) of *Lactobacillus plantarum* DMR14. (A) Fresh banana at 0 h, (B), and (C) after 3 days without treatment and with treatment, respectively.

The aim of this study was to investigate the impact of LAB on the shelf life of selected fruits when stored under laboratory conditions at room temperature (approximately 37 °C). To assess the extension of fruit shelf life, the cell-free supernatants (CFSs) of *Lactobacillus plantarum* DMR14 were applied to the fruits. Throughout a 7-day observation period, whole apples treated with CFSs remained fresh and free from contamination (Fig. 4 A, B, and C). Similarly, in the case of chopped apples, the treated samples exhibited better freshness compared to the untreated ones after 3 days (Fig. 5 A, B, and C). The experiment was also conducted with grapes over a period of 5 days (Fig. 6 A, B, and C) and with bananas over 3 days, yielding similar positive outcomes. These findings indicate that the CFSs of the LAB possess the potential to serve as a natural preservative (Fig. 7 A, B, and C).

### 3.11 Preservation of sugarcane juice

#### 3.11.1 Changes in pH levels with fermentation

**Fig. 8:**
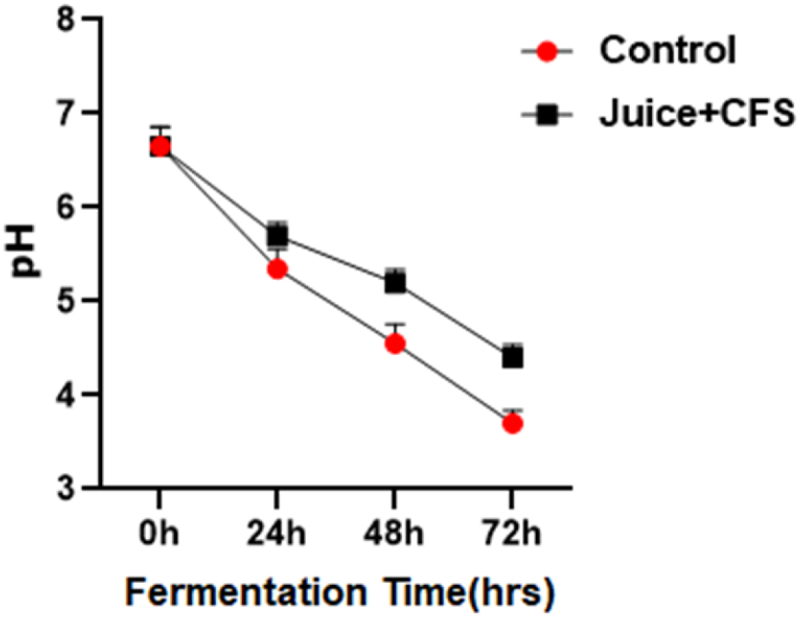
Change in pH levels with the fermentation of sugarcane juice with 100 μl of cell free supernatants (CFS) of the *Lactobacillus plantarum* DMR14. Data were recorded 24 h of interval.

The pH levels of the test samples were measured and are presented here in Fig. 8. The level of pH decreased with the passing of time due to the increase of the *Lactobacillus plantarum* DMR14. Initially, the pH was 6.5 and, by the end, the final pH had reached 3.8 for the control and 4.8 for the CFS-treated juice.

#### 3.11.2 Cell viability of LAB cultures during fermentation

**Fig. 9:**
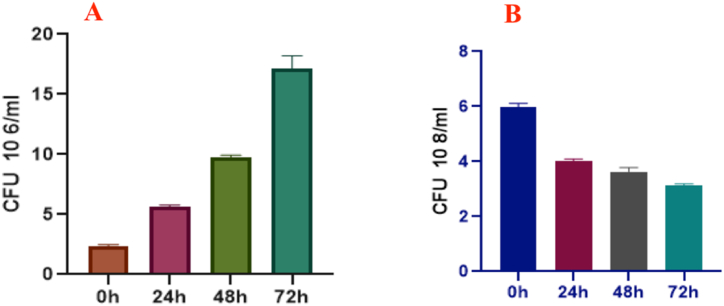
Viability of bacteria on CFS treated juice. (A) Lactic acid bacteria, (B) coliform bacteria. Data were recorded in various time durations (0 h, 24 h, 48 h, and 72 h).

Fermentation of sugarcane juice with lactic acid bacteria may have beneficial health effects on the body due to metabolites produced by the bacteria. With the passing of time, the concentration of the lactic acid bacteria increased gradually, indicating that fermented sugarcane juice could be a good career of LAB (Fig. 9A and Fig. S2). Furthermore, *E. coli* levels decreased, indicating that *Lactobacillus plantarum* DMR14 has anti-bacterial efficacy (Fig. 9B and Fig. S3).

## 4 Discussion

Lactic acid bacteria (LAB) are widely recognized as safe microorganisms, with the United States Food and Drug Administration (FDA) classifying them as generally recognized as safe (GRAS) and the European Food Safety Authority (EFSA) considering them to be under the qualified presumption of safety (QPS) (EFSA). Given their safety status, LAB has become an attractive option for food biopreservation as an alternative method for preventing the growth of pathogenic microorganisms. Due to their competitive edge against pathogenic bacteria, LAB is ideal candidates for developing bioprotective agents for fresh fruits, vegetables, and fruit juice. This natural preservation approach not only increases the shelf life of these perishable food products but also helps to maintain their nutritional content while eliminating the need for synthetic preservatives that may have adverse effects on human health and the environment [7,46]. In an effort to identify new sources of probiotic LAB for the preservation of fruits and fruit juice, the recent present study focused on isolating LAB from fermented oats. The study aimed to demonstrate the potential of cereal-based meals as a source of probiotic LAB, particularly for individuals who are unable to consume dairy-based products due to lactose intolerance or allergies. One of the key objectives of the study was to evaluate the probiotic potential of the isolated LAB strains and their ability to preserve fruits and fruit juice naturally. The findings suggest that the LAB strains isolated from fermented oats have the potential to be used as probiotics and natural preservatives, providing a promising alternative to conventional preservatives. By utilizing a non-dairy source of probiotic LAB, this approach could provide an option for individuals with dietary restrictions while promoting sustainable and healthier food preservation practices. In this study, *Lactobacillus plantarum* DMR14 was isolated from fermented oats using MRS selective media and was identified through 16S rRNA gene sequencing (as shown in Fig. 1). To determine its potential as a probiotic strain, the isolate was subjected to various criteria, including antagonistic efficacy, pH tolerance, bile salt tolerance, antibiotic susceptibility, among others. Among these criteria, pH tolerance is a crucial factor for determining the viability of probiotic strains. Probiotic bacteria should have the ability to survive in the highly acidic environment of the stomach and tolerate low pH values as low as pH 2.0 [47]. Thus, the pH tolerance *of L. plantarum* DMR14 was evaluated as part of the study's objective to assess its probiotic potential. Here, *Lactobacillus plantarum* DMR14 showed a wide range of pH tolerance (ranging from 2 to 7), as well as bile salt resistance, indicating that it has wide range of pH tolerance. Furthermore, another most important characteristic of probiotics is resistance to bile salts since these substances breakdown membrane lipids, which results in cell leakage and death. The acid and bile salt tolerance for the LAB varied significantly due to the specific nature of the strain, and thus depended on species. In previous studies, *L. plantarum* and *E. lactis* showed acceptable survival in a bile salt environment [43] even in a high concentration (0.3%). In this study, by being tolerant to high bile salt concentration (0.3%) the isolated strain full filled other criteria of the probiotic strains.

Antibiotic resistance is one of the key parameters for considering any microbe to be safe [48]. However, the genetic mechanisms for antibiotic susceptibility in LAB strains are limited. There are several genes in several species of the LAB that are responsible for antibiotic resistance [49]. *Lactobaccilus* are usually sensitive to the cell wall-targeting penicillin and susceptible to antibiotics that inhibit protein synthesis, such as chloramphenicol, lincomycin and tetracyclin. Here, *Lactobacillus plantarum* DMR14 was found to be resistant against amoxicillin (AMX) and erythromycin (E) and showed intermediate resistance to penicillin (P) and ampicillin (AMP). From this result, it is clear that antibiotic susceptibility and resistance of LAB is also varying with different species. Overall, the findings suggest that *L. plantarum* DMR14 exhibits promising probiotic properties and has the potential to be used as a natural preservative for fruits and fruit juice.

Hemolysis activity testing is one of the necessary probiotic assays. The gamma-hemolytic potentiality of *Lactobacillus plantarum* DMR14 indicated that it is not toxic to the environment or to individuals. Additionally, the strain's test results for lipolytic and proteolytic activity were both favorable. Similar results were observed by Hassan Barzegar regarding *Lactobacillus plantarum* DMR14 [50]. Antagonistic activity of the bacteria is generally tested to use it as a natural killer of the pathogenic bacteria. Here, *Lactobacillus plantarum* DMR14 showed strong inhibition activity against *Shigella boydii* and moderate antagonistic efficacy against *Pseudomonas* sp., *Aeromonas* sp., and *Staphylococcus aureus*. Antagonistic activity of the strain was also observed in previous studies, which is in support of our outcomes [51,52]. Biofilm formation is one of the main causes of antibiotic resistance [53]. *Lactobacillus plantarum* DMR14 showed positive results for biofilm inhibition potential against all selected strains. Like in the antagonistic testing, the most prominent result in this case was also observed against *Shigella boydii*. Hojjatolah Zamani [54] also reported *Lactobacillus plantarum* DMR14's biofilm inhibition efficacy against *Staphylococcus aureus,* and *Escherichia coli*, which is consistent with our findings. Natural preservatives made from the healthy *Lactobacillus plantarum* DMR14 strain are particularly sought after because chemical preservatives are harmful to our health. Bacteriocin is a product of LAB that primarily serves as a preservative. Additionally, the human body's proteolytic enzymes can degrade bacteriocin, making this bacterial treatment safe for our health [55].

After confirming bacterial probiotic potentiality, the main applied sector of the work was to use it as a natural preservative for the fruit industries. So, in this study, the potential of *Lactobacillus plantarum* DMR14 to increase the shelf life of fruits was investigated by immersing apples in its CFS. The LAB strain was found to effectively prevent the rotting of apples even after seven days of storage. Sliced apples that were untreated turned yellow and dried out, while the coated ones maintained their original color and freshness. The same positive results were observed with bananas and grapes. The findings of this study were consistent with the results of previous research conducted by S. Agriopoulou et al. and Fang et al. who also used different strains of bacteria, including *Salimicrobium*, *Bacillus, Paenibacillus, Lactobacillus delbrueckii* subsp. *bulgaricus*, and *Leuconostoc lactis*, to preserve fruits such as apples, bananas, strawberry and grapes [[56], [57], [58]]. Additionally, it has been shown that *Lactobacillus equigeneosi* and *Bacillus Smithii* are effective at increasing the shelf life of pomegranates [45]*.*

In a trial investigation, sugarcane juice, one of the popular street juices in the country, was used as a medium to assess the suitability of LAB as a natural preservative. The study aimed to determine whether the CFS of *Lactobacillus plantarum* DMR14 could be used as a powerful preservative for the juice. The results showed that the pH of the treated juice declined gradually compared to the control, while the concentration of LAB steadily rose, indicating that LAB could be used as a natural preservative for sugarcane juice. Similar results for orange juice and tomato juice were found in another study [59]. Qiao, Huahua, demonstrated that the sugarcane juice treated with *Lactobacillus* HNK10 and cultured at 37 °C for 48 h contained a significant amount of LAB, as well as high-quality and potent antibacterial activity, which supports our findings [60]. In our research, we also observed that CFU coliform bacteria in the juice treated with CFS dropped steadily. This result indicates that harmful bacteria declined as LAB increased. Thus, the CFS of *Lactobacillus plantarum* DMR14 may be employed in juice industries**.** However, no studies were found in which the researchers attempted to measure the concentration of pathogenic bacteria in LAB-fortified juice.

## 5 Conclusion

In these study, the probiotic lactic acid bacteria (LAB) from fermented oats was isolated to address the demand for natural preservatives. Here, *Lactobacillus plantarum* DMR14 strain showed strong antimicrobial and antibiofilm activity against *Shigella boydii*. Moreover, the strain's cell-free supernatant (CFS) extended the shelf life of the treated fruits and sugarcane juice. Here, the number of coliform bacteria decreased, while lactic acid bacteria increased in the CFS-treated sugarcane juice. The findings suggest *Lactobacillus plantarum* DMR14 as a potential fruit preservative in the food industry. This research contributes to the search for safer alternatives to chemical preservatives. Further exploration of probiotic-based preservation methods can benefit human health and the environment.

## Author contribution statement

Shirmin Islam, Md. Abu Saleh, Shahriar Zaman: Conceived and designed the experiments; Performed the experiments; Wrote the paper. Suvro Biswas, Tabassum Jabin, Md. Moniruzzaman, Jui Biswas: Performed the experiments; Analyzed and interpreted the data; Wrote the paper. Md. Salah Uddin, Md. Akhtar-E-Ekram, Abdallah M. Elgorban, Gajanan Ghodake, Asad Syed: Contributed reagents, materials, analysis tools or data; Wrote the paper.

## Data availability statement

Data will be made available on request.

## Funding

This work was supported by the Grants for Advanced Research in Education (Grant Number: LS20222099), 10.13039/501100004567Ministry of Education, Government of the People's Republic of Bangladesh. It was also supported by the Researchers Supporting Project Number (RSP2023R56), 10.13039/501100002383King Saud University, Riyadh, Saudi Arabia.

## Declaration of competing interest

The authors declare that they have no known competing financial interests or personal relationships that could have appeared to influence the work reported in this paper.
