# Supplementary material for: Probiotic potential of Lactobacillus plantarum DMR14 for preserving and extending shelf life of fruits and fruit juice
Source: Heliyon. 2023 Jun 19;9(6):e17382. doi: 10.1016/j.heliyon.2023.e17382 (PMC10361358; doi:10.1016/j.heliyon.2023.e17382)
Supplement: Multimedia component 1 [file mmc1.docx]

Probiotic potential of *Lactobacillus plantarum* DMR14 for preserving and extending shelf life of fruits and fruit juice

Shirmin Islam^1^, Suvro Biswas^1^, Tabassum Jabin^1^, Md. Moniruzzaman^1^, Jui Biswas^1^, Md. Salah Uddin^1^, Md. Akhtar-E-Ekram^1^, Abdallah M. Elgorban^2^, Gajanan Ghodake^3^, Asad Syed^2^, Md. Abu Saleh^1,*^, Shahriar Zaman^1.*^

**^1^**Microbiology Laboratory, Department of Genetic Engineering and Biotechnology, University of Rajshahi, Rajshahi-6205, Bangladesh.

^2^Department of Botany and Microbiology, College of Science, King Saud University, P.O. Box 2455, Riyadh 11451, Saudi Arabia.

**^3^**Department of Biological and Environmental Science, Dongguk University-Seoul, Ilsandong-gu, Goyang-si, 10326, Gyeonggi-do, South Korea.

***** Correspondence: szaman@ru.ac.bd; saleh@ru.ac.bd

**Table S1:** List of antibiotics used for antibiotic sensitivity test in this study.

| **Antibiotics Name** | **Per Disc Concentration** |
| --- | --- |
| Kanamycin (K) | 30 |
| Gentamycin (G) | 10 |
| Penicillin (P) | 10 |
| Ampicillin (AMP) | 25 |
| Ciprofloxacin (CIP) | 5 |
| Amoxicillin (AMX) | 30 |
| Doxycycline (DO) | 30 |
| Chloramphenicol (C) | 30 |
| Erythromycin (E) | 15 |

**Table S2:** List of bacterial strains used for antimicrobial test.

| **Sl. no.** | **Name of the used bacterial strains** |
| --- | --- |
| **01** | *Shigella boydii* |
| **02** | *Pseudomonas* sp. |
| **03** | *Staphylococcus aureus* |
| **04** | *Escherichia coli* |
| **05** | *Bacillus cereus* |
| **06** | *Aeromonas* sp. |


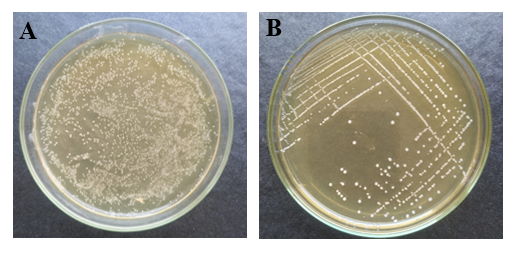


**Fig. S1:** Isolation of LAB on MRS agar media. (A) The spreading plate of the LAB and (B) streaking plate of the LAB on MRS agar.


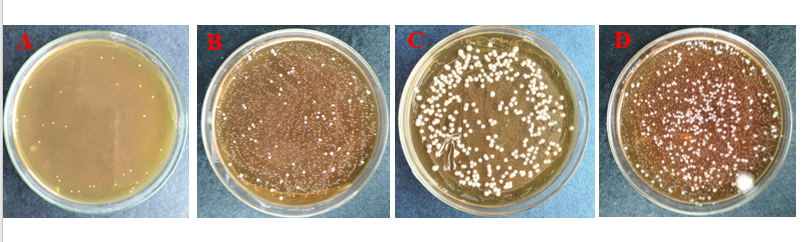


**Fig. S2:** Viability of the lactic acid bacteria on fermented juice on MRS agar


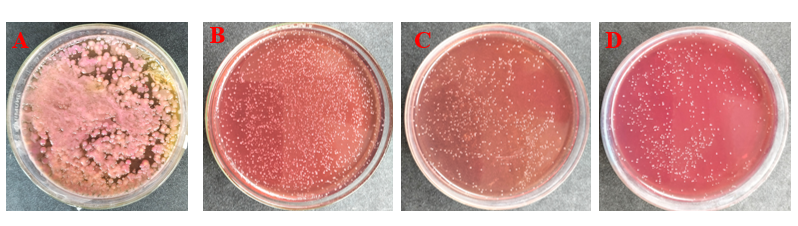


**Fig. S3:** Viability of the coliform bacteria on MacConkey agar
